# Left and Right Amygdala - Mediofrontal Cortical Functional Connectivity Is Differentially Modulated by Harm Avoidance

**DOI:** 10.1371/journal.pone.0095740

**Published:** 2014-04-23

**Authors:** Chris Baeken, Daniele Marinazzo, Peter Van Schuerbeek, Guo-Rong Wu, Johan De Mey, Robert Luypaert, Rudi De Raedt

**Affiliations:** 1 Department of Psychiatry and Medical Psychology, Ghent University, Ghent, Belgium; 2 Department of Psychiatry, University Hospital (UZBrussel), Brussels, Belgium; 3 Ghent Experimental Psychiatry (GHEP) Lab, Ghent, Belgium; 4 Department of Data Analysis, Ghent University, Ghent, Belgium; 5 Department of Radiology and Medical Imaging, University Hospital (UZBrussel), Brussels, Belgium; 6 Key Laboratory for NeuroInformation of Ministry of Education, School of Life Science and Technology, University of Electronic Science and Technology of China, Chengdu, China; 7 Department of Experimental Clinical and Health Psychology, Ghent University, Ghent, Belgium; Bellvitge Biomedical Research Institute-IDIBELL, Spain

## Abstract

**Background:**

The left and right amygdalae are key regions distinctly involved in emotion-regulation processes. Individual differences, such as personality features, may affect the implicated neurocircuits. The lateralized amygdala affective processing linked with the temperament dimension Harm Avoidance (HA) remains poorly understood. Resting state functional connectivity imaging (rsFC) may provide more insight into these neuronal processes.

**Methods:**

In 56 drug-naive healthy female subjects, we have examined the relationship between the personality dimension HA on lateralized amygdala rsFC.

**Results:**

Across all subjects, left and right amygdalae were connected with distinct regions mainly within the ipsilateral hemisphere. Females scoring higher on HA displayed stronger left amygdala rsFC with ventromedial prefrontal cortical (vmPFC) regions involved in affective disturbances. In high HA scorers, we also observed stronger right amygdala rsFC with the dorsomedial prefrontal cortex (dmPFC), which is implicated in negative affect regulation.

**Conclusions:**

In healthy females, left and right amygdalae seem implicated in distinct mPFC brain networks related to HA and may represent a vulnerability marker for sensitivity to stress and anxiety (disorders).

## Introduction

Emotions involve brain networks including (pre)frontal cortical and limbic areas [1], [2]. Within these emotional networks the amygdalae play a crucial role [3], [4]. Biologically oriented theories suggest specific affective information-processing roles for the left and the right amygdala [5], [6]. An emotional stimulus automatically activates the right amygdala, which is thought to play a role in dynamic emotional stimulus detection, while the left amygdala seems to be more involved in specific, sustained stimulus evaluation [7], [8]. However, how individual differences can affect left and right amygdala related neurocircuits differently remains poorly understood [9]–[12]. Trait and state anxiety has been found to modulate amygdala resting-state functional connectivity (rsFC) related to ventromedial prefrontal cortical (vmPFC), but not with the dorsomedial prefrontal cortical (dmPFC) activity [13]. Together with the amygdalae these brain regions are thought to be involved in the neuronal circuits of fear behavior, in self-referential processing and social interactions [14], [15]. Furthermore, it has been suggested that in order to stop the generation of anxious states the strength of amygdala–mPFC functional connectivity during rest represents efficient crosstalk between these brain regions [16], [17].

Only recently, researchers became interested in the relationship of amygdala rsFC with personality features [18], such as Harm Avoidance (HA). Cloningers' psychobiological theory on personality and genetic inheritance states that scoring high on the temperament factor HA is related to increased behavioral inhibition and implies a genetically determined bias towards being cautious, apprehensive and overly pessimistic [19]. Healthy individuals scoring high on HA are more at risk for developing mood- and anxiety disorders in the course of their lives [20], [21]. Based on anatomical parcellations of the amygdalae, Li and colleagues [22], have reported on sex-related amygdala rsFC differences in relation to HA. In spite that functional imaging data point to lateralization differences in amygdala emotional functioning in healthy participants, with especially the left amygdala implicated in negative affect [6], and a major topic in our line of research [12], [23]–[25], to date it remains unclear whether the temperament dimension HA may affect left or right amygdala rsFC in relation to the mPFC differently. Brain imaging approaches such as resting-state fMRI combined with HA measurements may increase our understanding of how behavioral more inhibited individuals with the tendency to be more pessimistic could be at higher risk to develop affective disorders [17], [26].

Consequently, the aim of the current study is to test the hypothesis that in a homogeneous sample of females - never documented to have suffered from neuropsychiatric illnesses – individual scores on HA are related to differential left and right amygdala - mPFC coupling. Importantly, the selection of the left and right amygdala nodes was based on brain anatomical coordinates provided by a neuroimaging study of emotion processing and emotion regulation in women resilient or susceptible to the depressogenic effects of early life stress [27]. These nodes fall within the area referred to as the Superficial Amygdala (SA), not surprisingly reported to be involved in the processing of social information [28], [29] and especially relevant to Harm Avoidance.

Across all subjects, we hypothesized the existence of rsFC differences for the connections of left and right amygdala with distinct regions in the brain. We hypothesized in high HA scoring females stronger rsFC correlations between predominantly the left amygdala seed and the mPFC. Within the mPFC, we expected in particular left amygdala rsFC-HA correlations with the vmPFC. Because amygdala lateralization differences are not consistently reported for the dmPFC, we hypothesized no lateralized amygdala rsFC-HA correlations with dmPFC areas.

## Methods and Experimental Procedures

### 1. Participants

The study was approved by the ethics committee of our University Hospital (UZBrussel) and in accordance with the guidelines laid down in the declaration of Helsinki (2004). All participants gave written informed consent. This study was part of a larger project investigating several neuro-cognitive markers in affective disorders. After the structural MRI, all participants went through the rs-fMRI. Hereafter other psychological imaging paradigms were performed, not related to the current study.

Sixty right-handed female individuals (mean age = 21.7 y, sd = 2.5), all university students, were recruited. Right-handedness was assessed with the van Strien questionnaire [30]. Because besides gender also age may confound rsFC results, all participants were selected within a narrow age range [31]. Participants taking medication, other than birth-control pills, were excluded. None of the participants reported to have ever used psychotropic medications such as antidepressants, mood stabilizers or antipsychotics, and all were free of illicit drugs. To exclude psychiatric or neurological diseases, all volunteers were screened by the first author (C.B). Psychiatric disorders were assessed by the Dutch version of the Mini-International Neuropsychiatric Interview (MINI) [32]. Participants with a psychiatric disorder and/or a score higher than eight on the Beck Depression Inventory (BDI-II [33]) were excluded.

### 2. Temperament and Character Inventory

The Temperament and Character Inventory (TCI) is a 240-item questionnaire developed by Cloninger and colleagues [19], [34]. The questionnaire is based on a psychobiological model that aims to explain individual differences in personality traits [35]. The TCI consists of 4 temperament scales (Harm Avoidance (HA), Novelty seeking (NS), Reward dependence (RD), Persistence (P)), and three character scales (Cooperativeness (CO), Self-directedness (SD) and Self Transcendence (ST)) [34]. This inventory has been used in a variety of studies examining psychobiological substrates of personality, including neurobiological, neuroimaging and genetic methods [12], [36], [37]. We extracted only the temperament dimension HA for our purposes (minimum score = 0, maximum score is 36).

### 3. Scanning Procedure

During the resting state measurements, involving exactly five minutes of scanning, all participants were asked to stay awake with their eyes closed and to think of nothing in particular. To reduce sensory confounds as much as possible, the light in the room was dimmed during scanning. After the scan, the participants were asked to confirm that they had been awake throughout the scan and had complied with the instructions. All resting state fMRI scans were performed on Monday afternoons, between 3:00 pm and 6:00 pm.

All scans were performed on a 3T Philips Achieva MRI system (Philips, Best, The Netherlands) with an eight channel SENSE head coil. fMRI measurement was done using a SE-EPI sequence (TR/TE = 3000/70 ms; Flip angle = 90°; FOV = 230×230 mm^2^; resolution = 1.80×1.80 mm^2^; Slice thickness/gap = 4.00/1.00 mm; number of slices = 24; number of dynamics = 100; dynamic time resolution = 3000 ms). After the fMRI scan a 3D anatomical scan using a 3D T1 TFE sequence (TR/TE = 12.00/3.71 ms; Flip angle = 10°; FOV = 240×240×200 mm^3^; resolution = 1.00×1.00×2.00 mm^3^; number of slices = 100) was performed, yielding an anatomical underlay for the fMRI results.

The fMRI data were analyzed with the SPM8 software (Wellcome Department of Cognitive Neurology, London, UK). Slice-time correction was performed to correct for small differences in the time offset of consecutively measured slices. Hereafter, the images were realigned to the first volume of the time series in order to correct for head movements. Subsequently, all fMRI brain volumes were normalized to the EPI MNI template; resampled to 3-mm isotropic voxels and spatially smoothed using an 8-mm full-width half-maximum Gaussian kernel. The anatomical scans were normalized to the T1 MNI template.

Several further processing steps preceded the voxel-based correlation analysis. Data were linearly detrended and band-pass filtered (0.01–0.08 Hz). Spurious or nonspecific sources of variance were removed from the data through linear regression of: 1) the six head-motion parameters obtained in the realigning step, 2) the signal from a region in the cerebrospinal fluid, 3) the signal from a region centered in the white matter. As proposed by Murphy et al. [38] and Weissenbacher et al. [39] resting state data were processed without global signal regression. Correlation maps were obtained by extracting the BOLD time course from a seed region, then computing the correlation coefficients characterizing the correlations between that time course and the time courses from all other brain voxels. The seed regions were 6-mm-diameter spheres designed to encompass the left (MNI coordinates x = −20, y = −4, z = −15) or right amygdala (x = 22, y = −2, z = −15). These MNI coordinates were selected following the recent paper of Cisler et al [27]. To combine results across subjects and compute statistical significance, Fisher's r-to-Z transformation was used to convert these correlation maps into Z maps (maps quantifying local ‘rsFC strength’, or simply ‘rsFC’). The Z maps were submitted to a random-effects analysis in SPM8. A one-sample *t*-test containing age as covariate was performed for the left and right amygdala rsFC separately. To evaluate significant differences between left and right amygdala rsFC, a paired *t*-test was performed with age as covariate. All analyses used a cluster significance level of *p*<0.05, corrected for multiple comparisons (Family Wise Error (FWE)). We listed all significant clusters with a cluster extent threshold (K) of at least 50 voxels.

Concerning the influence of the temperament dimension HA on left and right amygdala rsFC separately, we calculated Pearson's correlation coefficients between the Fisher-*z*-transformed rsFC strength and HA scores for each voxel, producing another set of *r*-maps. To examine our primary research question; the influence of HA on lateralized amygdala rsFC, we calculated the Pearson's correlation coefficient between the difference of the Fisher-z-transformed rsFC strength and HA scores for each voxel left vs. right amygdala rsFC. After Fisher-z transformation on *r*-maps, we mapped the voxels with *p*-values<0.05. The anatomical labels and Montreal Neurological Institute (MNI) coordinates were obtained by the xjView MATLAB toolbox (http://www.alivelearn.net/xjview).

## Results

The range in HA scores was between 2 and 30 (mean HA score = 15.52, sd = 7.04). The Shapiro-Wilk normality test showed that HA scores were normally distributed (*p* = .46). No volunteer stated to have fallen asleep during scanning. Due to exceeding 1.5 mm and 1.5 degree in maximum head motion, four female volunteers were removed from rs-fMRI analyses, leaving a total of 56 participants.

### 1. Amygdala rsFC

#### 1.1. Left amygdala rsFC

**Figure 1:**
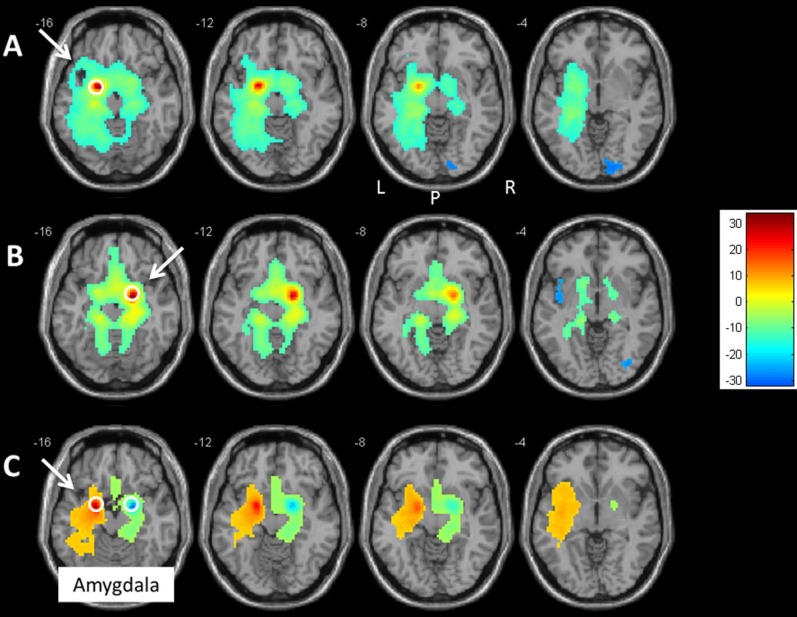
Left and right amygdala rsFCs. Transversal slides displaying the results of A) the one-sample t-test for the left-amygdala rsFC and B) the one-sample *t*-test for the right-amygdala rsFC. Colors from yellow to red represent significantly stronger FC and colors from green to blue represent the opposite. C) the comparison of the rsFCs of left vs. right amygdala the paired *t*-test between the rsFCs of the left and right amygdala seeds. Colors from yellow to red represent significantly stronger FC with the left than with the right amygdala. Colors from green to blue represent the opposite: significantly stronger FC with the right amygdala than with the left. The amygdala seeds are displayed by a white circle. For an overview of all significant clusters see Table 1. P = posterior, L = left, R = right.

See Fig. 1 A. The result of the one-sample *t*-test for the left-amygdala rsFC showed one large significant cluster in the left parahippocampal gyrus (K = 6285; MNI coordinates: x = −18, y = −3, z = −15). On the left hemisphere, this rsFC region included hippocampus, insula and subgenual anterior cingulate cortex (sgACC), putamen and claustrum, fusiform gyrus and culmen. This cluster also extended to the right hippocampus, the left and right gyrus rectus, and the thalamus.

In addition, the one-sample *t*-test showed a significant inverse correlation between the left amygdala and the right middle occipital gyrus (BA 18: K = 392; MNI coordinates: x = 15, y = −87, z = 6).

#### 1.2. Right amygdala rsFC

The result of the one-sample *t*-test for the right-amygdala rsFC showed two significant clusters. One large cluster was situated in the right parahippocampal gyrus (K = 3376; MNI coordinates: x = 18, y = −3, z = −15). A second cluster was located in the right anterior cingulate cortex (BA 24; K = 148; x = 3, y = 27, z = 18). See also Fig. 1 B.

The one-sample *t*-test revealed an inverse correlation between the right amygdala and the right fusiform gyrus (K = 89; x = 51, y = −18, z = −30), the left insula (BA 13; K = 62; x = −42, y = −6, z = 0), the left middle frontal gyrus (BA 9; K = 410; x = −33, y = 33, z = 42), and the right (K = 222; x = 48, y = −66, z = 6) and left middle temporal gyrus (K = 186; x = −45, y = −69, z = 9).

#### 1.3. Comparison between the left and right amygdala rsFCs

See also Fig. 1 C. The paired *t*-test revealed that the contrast (left amygdala rsFC>right amygdala rsFC) yielded a significant cluster in the left parahippocampal gyrus, with the maximum peak in the left amygdala (K = 2889; MNI coordinates: x = −21, y = −3, z = −15). Other peak areas were located at the left side: in the hippocampus, insula (BA 13), putamen, fusiform gyrus, and pons.

The paired *t*-test for the contrast (right amygdala rsFC>left amygdala rsFC) revealed a significant cluster one in the right parahippocampal gyrus with the maximum peak in the right amygdala (K = 578; x = 21, y = −3, z = −15), hippocampus, culmen, and lingual gyrus (BA 17).

### 2. Amygdala rsFC-HA correlation analyses

#### 2.1. Left amygdala

**Figure 2:**
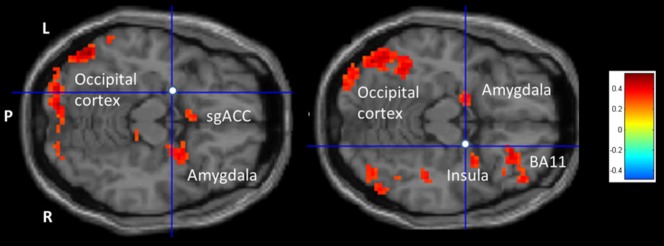
Amygdala rsFC -HA correlation analysis. Transversal slide exhibiting positive (yellow to red) and negative (green to blue) correlation clusters for the correlation between the rsFC of the left amygdala seed (Left crosshair on white sphere; MNI coordinates: x = −20, y = −4, z = −15), the right amygdala seed (Right crosshair on white sphere; MNI coordinates: x = 22, y = −2, z = −15), and HA. For an overview of all significant clusters see Table 1 and Table 2. P = posterior, L = left, R = right, BA = Brodmann area.

The results of the rsFC-HA correlation analysis for the left amygdala seed (MNI coordinates x = −20, y = −4, z = −15) yielded a positive association in the left occipital gyrus and the sgACC (BA 25), the right amygdala and larger parts of the cerebellar regions. Negative correlations were observed in the left cerebellum and the right superior frontal gyrus (BA 9). See Table 1 and Fig. 2.

**Table 1:** Results for the correlation between the individual scores on Harm Avoidance and the rsFC of the left amygdala seed.

Seed	Correlation	Hemisphere	Cluster size	Anatomical region	BA	Z-value	Peak coordinates (x,y,z) (mm)
**Left Amygdala**							
	**Positive correlation**						
		**Left**	242	Inferior occipital Gyrus	-	0.53	−24 −93 −12
			126	Middle occipital gyrus	-	0.50	−51 −66 −15
			60	Gyrus Rectus	25	0.43	−3 12 −21
		**Right**	236	Cerebellum posterior lobe	-	0.50	18 −48 −36
			106	Inferior frontal gyrus	45	0.45	63 12 24
			91	Limbic lobe	amygdala	0.49	24 6 −21
			71	Parietal lobe	39	0.47	45 −69 30
	**Negative correlation**						
		**Left**	59	Cerebellum	-	−0.42	−36 −66 −24
		**Right**	64	Superior Frontal Gyrus	9	−0.42	18 48 39

For each cluster, we reported the Z-value and MNI coordinates at the position of the maximum, the cluster size (K) and the corresponding Brodmann area (BA).

#### 2.2. Right amygdala

The rsFC-HA correlation analysis for the right amygdala seed (MNI coordinates: x = 22, y = −2, z = −15) showed a positive association with the left inferior frontal gyrus (BA 10) and the post cinglulate gyrus (BA 31). Strong correlations were also observed in the right middle frontal gyrus (BA 9 as well as BA 11), the right parietal lobe (BA 7), and cerebellar regions. Bilateral positive correlations were observed for the parietal and occipital cortex and thalamus. Negative correlations were found in the left ACC (BA 24), the right parahippocampus and cerebellum bilaterally. See Table 2 and Fig. 2.

**Table 2:** Results for the correlation between the individual scores on Harm Avoidance and the rsFC of the right amygdala seed.

Seed	Correlation	Hemisphere	Cluster size	Anatomical region	BA	Z-value	Peak coordinates (x,y,z) (mm)
**Right Amygdala**							
	**Positive correlation**						
		**Left**	232	Middle Occipital Gyrus	18	0.48	−45 −72 −12
			232	Parietal Lobe	Precuneus	0.43	−27 −69 42
			57	Parietal Lobe	42	0.39	−60 −27 12
			53	Parietal lobe	40	0.34	−27 −24 57
			136	Thalamus	-	0.37	−15 −18 15
			71	Post cingulate gyrus	31	0.43	0 −54 30
			66	Inferior Frontal Gyrus	10	0.47	−45 30 3
			58	Parahippocampal gyrus	Amygdala	0.49	−9 0 −24
		Right	307	Occipital lobe	19	0.48	24 −72 −6
			306	Cerebellum posterior lobe	-	0.51	15 −57 −45
			256	Postcentral Gyrus	-	0.54	33 −21 39
			136	Parietal Lobe	7	0.47	24 −54 60
			132	Inferior temporal lobe (Fusiform gyrus)	-	0.47	54 −18 −24
			117	Thalamus	-	0.47	18 −24 12
			84	Limbic lobe	30	0.44	21 −51 0
			79	Middle Frontal Gyrus	9	0.39	45 21 36
			76	Middle frontal gyrus	11	0.41	27 33 −15
			72	Superior temporal gyrus	38 (amygdala)	0.36	42 3 −24
	**Negative correlation**						
		**Left**	23	Cuneus	-	−0.32	−12 −78 6
			49	Anterior Cingulate Cortex	24	−0.41	−6 21 27
		**Right**	77	Parahippocampal gyrus	30	0.41	9 −39 −3
			45	Cerebellum	-	−0.39	3 −81 −21

For each significant cluster, we reported the Z-value and MNI coordinates at the position of the maximum, cluster size (K) and the corresponding Brodmann area (BA).

#### 2.3. Left vs. right amygdala confined the entire sample

**Figure 3:**
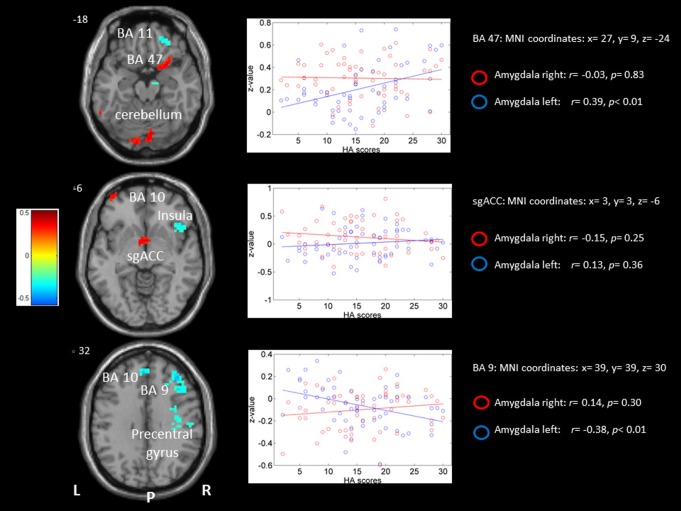
Left vs right amygdala rsFC -HA correlation analysis. Left column) Transversal slides displaying the results of the rsFC-HA correlation analyses for the left vs. right amygdala rsFC seeds. Colors from yellow to red represent significantly stronger FC with the left compared to the right amygdala. Colors from green to blue represent the opposite: significantly stronger FC for the right compared to the left amygdala. For an overview of all significant clusters see Table 3. P = posterior, L = left, R = right, BA = Brodmann area. Right column) Scatter plots representing left vs. right amygdala rsFC-HA correlations with their respective correlation coefficients. The red circles represent more left vs. right amygdala rsFC; the blue circles represent the reverse: more right vs. left amygdala rsFC.

rsFC-HA correlation analyses revealed that the high scorers on HA displayed stronger left compared to right amygdala rsFC within some clusters situated around the left premotor cortex (BA 6) and supplementary motor area (SMA). Further, healthy females scoring higher on HA displayed stronger left vs. right amygdala rsFC in the right inferior frontal gyrus and the right sgACC (BA 25). See Table 3 and Fig. 3.

**Table 3:** Results for the correlation between the individual scores on Harm Avoidance and the rsFC of the left vs. right amygdala seed.

Seed	Hemisphere	Cluster size	Anatomical region	BA	Z-value	Peak coordinates (x,y,z) (mm)
**Left>Right Amygdala Correlation**						
	**Left**	81	Cerebellum posterior	-	0.40	−12 −81 −21
		55	Precentral gyrus	6	0.52	−18 −12 48
		35	Precentral gyrus	6	0.45	−12 −21 75
		21	Supplementary motor area	6	0.42	−15 −3 66
		22	Middle frontal gyrus	10	0.34	−39 60 −6
	**Right**	63	Inferior frontal gyrus (Parahippocampus)	47	0.49	27 9 −24
		21	Anterior cingulate	25	0.37	3 3 −6
		21	Inferior frontal gyrus	45	0.36	60 24 24
**Right>Left Amygdala Correlation**						
	**Left**	47	Posterior cingulate	30	0.38	−3 −54 12
		30	Medial frontal gyrus	10	0.40	−9 51 15
	**Right**	228	Middle frontal gyrus	9	0.52	39 39 30
		162	Precentral gyrus	**-**	0.57	33 −21 42
		49	Inferior frontal gyrus	Insula	0.36	42 24 −6
		29	Fronterior superior orbital gyrus	11	0.39	24 42 −15
		25	Parahippocampal gyrus	-	0.35	18 −12 −24

For each cluster, we reported the Z-value and MNI coordinates at the position of the maximum, the cluster size (K) and the corresponding Brodmann area (BA).

On the other hand, when comparing right vs. left amygdala rsFC, higher scores on HA showed stronger rsFC-HA correlation within the right parahippocampal gyrus and prefrontal cortex, including the middle frontal gyrus (BA 9), insula, and orbitofrontal cortex (BA 11). In this contrast stronger left hemispheric rsFC-HA correlations were found in left posterior cingulate (BA 30) and medial prefrontal gyri (BA 10).

#### 2.4. Left vs. right amygdala confined to high HA scorers

**Figure 4:**
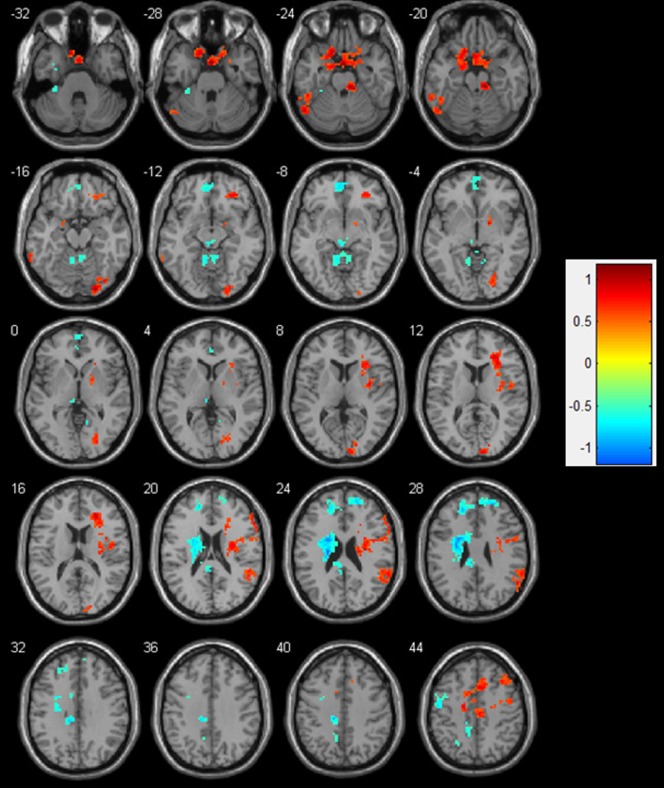
Left vs right amygdala rsFC -HA correlation analysis confined to high HA scoring females. Transversal slides displaying the results of the rsFC-HA correlation analyses for the left vs. right amygdala rsFC seeds. Colors from yellow to red represent significantly stronger FC with the left compared to the right amygdala. Colors from green to blue represent the opposite: significantly stronger FC for the right compared to the left amygdala. For an overview of all significant clusters see Table 4.

To evaluate possible involvement of HA in the development of affective disorders, we selected those females scoring high on HA according Dutch and Flemish normative data set (n = 1041) (The Netherlands are a neighboring country closely related to Flanders, Belgium). According this data set, which provide normative TCI data for males and females separately [40], from our 56 female participants 18 scored high or very high on the temperament dimension HA. For an overview see Table 4 and Fig. 4.

**Table 4:** Results for the correlation between the selected individual scores high on Harm Avoidance (n = 18) and the rsFC of the left vs. right amygdala seed.

Seed	Hemisphere	Cluster size	Anatomical region	BA	Z-value	Peak coordinates (x,y,z) (mm)
**Left>Right Amygdala Correlation**						
	**Left**	286	Limbic lobe	-	0.08	0 0 −24
		47	Cerebellum	-	2.17	−51 −63 −24
		30	Inferior temporal gyrus		0.98	−66 −45 −18
	**Right**	195	Medial frontal gyrus	6	1.56	48 0 51
		185	Insula	13	−1.35	36 −3 9
		181	Medial frontal gyrus	32/24	1.26	6 15 45
		127	Insula	13	0.77	27 33 15
		90	Occipital lobe	18	−4.41	9 −96 12
		79	Parietal lobe	40	−2.83	63 −48 24
		63	Occipital lobe	18	−3.09	24 −90 −15
		37	Middle frontal gyrus	11	−1.28	36 39 −12
		36	Middle frontal gyrus	8	2.00	36 24 45
		33	Cerebellum		1.09	12 −30 −21
		31	Inferior frontal gyrus	45	−2.94	60 24 21
		26	Lentiform nucleus	-	−0.60	18 0 −3
		20	Cerebellum	-	1.09	30 −66 −42
**Right>Left Amygdala Correlation**						
	**Left**	252	Caudate nucleus	-	1.32	−21 −15 24
		97	Cerebellum	-	3.32	−6 −51 −12
		92	Superior frontal gyrus	9	−0.28	−18 42 27
		89	Medial frontal gyrus	10/11	−0.003	−3 54 −9
		76	Cingulate gyrus	31	−0.30	−12 −27 39
		44	Precentral gyrus	6	1.22	−51 −9 45
		30	Precuneus	-	3.46	−12 −42 45
		28	Thalamus	-	−1.51	−3 −27 −9
		25	Parietal lobe	7	1.05	−24 −60 48
		21	Culmen	-	3.36	−33 −33 −30
		20	Inferior temporal gyrus	-	2.31	−33 3 −36
	**Right**	66	Superior frontal gyrus	10	−2.34	24 51 27
		24	Supplemental motor area	6	0.39	3 −12 78

For each cluster, we reported the Z-value and MNI coordinates at the position of the maximum, the cluster size (K) and the corresponding Brodmann area (BA).

In short, left vs. right amygdala rsFC showed stronger rsFC-HA correlations with the right hemisphere, such as the right insula, but importantly also with a larger cluster within the vmPFC, comprising both amygdalae and the sgACC, the inferior frontal and rectal gyrus, extending to both parahippocampi. This contrast also revealed significant rsFC-HA correlation with the dmPFC, more in particular the medial prefrontal gyrus (BA 32/24).

On the other hand, the right vs. left amygdala rsFC showed stronger rsFC-HA correlations with the left hemisphere, including the basal ganglia, the left superior frontal (BA 9) and bilateral medial frontal (BA 10) gyri.

## Discussion

Although not the main scope of the current research, our overall amygdala rsFC observations without the inclusion of the HA scores are in line with the findings of Roy and colleagues [41] where spontaneous activities in the amygdalae predicted spontaneous activity in similar parahippocampal and prefrontal regions, the thalamus, and occipital cortex. Our general rsFC results point to distinct functional network connections largely within the same hemisphere for left or right amygdala seed separately. Further, the amygdala rsFC-HA correlations showed mostly positive associations with temporal, parietal, occipital and cerebellar cortices. These areas play critical roles in the perceptual processing of socially and emotionally relevant visual information, especially in non-clinical samples with higher trait anxiety [42]–[46].

As hypothesized, left vs. right amygdala rsFC-HA correlation analyses showed that females scoring higher on HA displayed stronger left amygdala FC within mPFC regions. Within the vmPFC, more in particular the sgACC, this area is related to arousal processes and implicated in a corticolimbic neurocircuit associated with ‘visceromotor’ functions playing an important role in modulating affect, such as sadness activation and ruminative thought patterns [47]. This functional amygdala - sgACC coupling has also been reported in female anxiety patients scoring higher than controls on HA [48]. However, our findings seem to be in disagreement with the study of Kim and colleagues [13] where reverse amygdala –vmPFC FC results were reported in relation to higher scores on the State and Trait Anxiety Inventory self-report questionnaires (STAI-S, STAI-T [49]). Of note, albeit higher HA scorers may display higher anxiety levels, HA and STAI scales do not measure the same construct [20] (Cloninger et al., 2006). Further, although Kim et al [13] defined the sgACC as part of the vmPFC, on a functional level it may be that not the sgACC but the more ventral-rostral portions of the ACC and vmPFC are involved in regulating strong emotional responses [50]–[52]. Indeed, in the selected group of High HA scorers in particular the left amygdala and the right medial frontal gyrus (BA 32/24) were functionally connected, the latter pregenual anterior cingulate cortex (pACC) documented to control emotional neuronal processes, such as self-conscious emotion [53], [54].

The left vs. right amygdala rsFC-HA correlation analyses showed positive right amygdala rsFC-HA with predominantly the right hippocampus and insula, the dorsolateral prefrontal cortex (DLPFC) and orbitofrontal cortex (OFC). The latter are part of the more dorsal parts of the mPFC [17]. See also Table 3. In spite that we did not hypothesize lateralized amygdalae rsFC-HA correlations with dmPFC areas, these findings concur with a right prefrontal involvement in the regulation of negative affect. Besides that the DLPFC and the amygdala are indirectly implicated in top-down/bottom-up emotion-regulation processes [55], the right DLPFC in particular seems to be implied in behavioral inhibition, negative affect regulation, increased vigilance and sustained attention, uncertainty and ambiguity [56] (Shackman et al., 2009). Of interest, the right OFC shows co-activations with insular parts associated with interoception and gustation [57]. Interoceptive information such as visceral sensations implicated in processes of awareness and experiences of aversive responses are thought to be channeled into in the right anterior insula [58]–[60]. Indeed, the neurobiological modulation of stress responses has been reported to be lateralized to the right prefrontal cortex [61], [62]. Interestingly, in the selected group of high HA scorers, also the left amygdala showed significant FC with the right insular regions. Furthermore, the right DLPFC, OFC and insula were found to be activated in anticipation to withdrawal-related emotional experiences [63]. Our current results further provide insight that in behavioral more inhibited and cautious individuals, not only the right amygdala may play a key role in regulating these processes, but in more stress sensitive individuals both amygdalae seem to be involved. In addition, rsFC-HA correlations showed that the both amygdalae were significantly stronger functionally correlated with the medial frontal gyrus (BA 10) also part of the dmPFC area. These rostral parts of the dmPFC are associated with emotion regulation, sustained attention, memory, and mentalizing processes [64]–[67]. Being part of the DMN, the BA 10 is implicated when individuals make self-relevant affective decisions [68], [69]. Importantly, right amygdala also correlated with the posterior cingulate gyrus, part of the DMN as well, and together with the mPFC and hippocampus is thought to be implicated in the processing of autobiographical memory, past self-relevant stimuli and future prospection [70]. As this DMN is especially implicated when at risk for clinical depression, it is tempting to speculate that this right amygdala rsFC-HA dmPFC association may represent a ‘neuronal network vulnerability’ for the development of mood disorders in a later stage of life.

Finally, for the higher HA scorers bilateral amygdala rsFC-HA correlations extended from the vmPFC to the basal ganglia. This is an important observation because the involvement of dopaminergic nuclei is not surprising. Besides that the amygdala, hippocampus, and these ventromedial prefrontal cortical areas are key brain regions that not only modulate emotions and cognition but also the response to stress itself - resulting in hypertrophy of dendritic arborization and increases in spine density [71]–[73] - the mentioned vmPFC areas are consistently involved in positive and negative reward processing (for an overview see Liu et al. [74]). This is of particular importance in more behavioral inhibited and pessimistic individuals. These dopaminergic neurons coming from the ventral tegmental area (VTA) are crucial for the recognition of rewards and their consumption [75]. Again, as the selected amygdala nodes fall within the area referred to as the Superficial Amygdala (SA) which has been shown to be specialized in the processing of social information [28]–[29], our results add to the assumption that individuals scoring high on HA not only display more behavioral inhibition and pessimism, but may also be more vulnerable to stressful interpersonal experiences.

Although the selection of psychopathology-free female subjects can be considered a major advantage of the study, including only healthy women within a certain age range means that we cannot generalize our findings to other populations. Because no cardiac and respiratory data were collected during rs-fMRI, this should be noted as a limitation of our study. As it has been reported that the different subnuclei of the amygdalae may have specific functional connections with distinct parts of the brain [41], [76], by not examining dedicated seeds in these subnuclei, important information could have been missed. However, our main research objective was to examine rsFC differences in relation to specific left and right-sided amygdalar nodes which were documented to be involved in emotion regulation brain networks among individuals resilient or susceptible to the depressogenic effects of early life stress [27]. This makes the choice of these selected nodes particularly relevant in relation to personality features such as harm avoidance. And again these nodes fall within the area referred to as the Superficial Amygdala involved in social information processing [28]–[29]. Nevertheless, future research examining amygdala rsFC in relation to personality features may do well to include a larger number of seeds, comprising the different amygdalar subnuclei.

In conclusion, amygdala rsFC analyses in relation to individual differences in HA may prove to be a valid method to investigate behavioural inhibition and pessimism, possible risk factors for mental illness development. Our rsFC-HA results confirm the right amygdala's key role in right anterior hemisphere cross-talk in females who are likely more stress-sensitive. Furthermore, the combination of enhanced left amygdala –vmPFC and right amygdala-dmPFC coupling may represent a vulnerability marker for females with an elevated risk to develop mood and anxiety disorders. Longitudinal follow-up studies in both genders are needed to substantiate such hypotheses and to demonstrate whether or not such amygdala rsFC-HA patterns within the medial prefrontal cortex are of use to predict the development of mood and anxiety disorders.
